# Eczema at the Umbilicus

**Published:** 1881-02

**Authors:** 


### ECZEMA AT THE UMBILICUS.

CLOSE around the cicatrix is a red and irritated or eczematous patch of skin, while from the depths of the umbilical fossa oozes a thin purulent fluid. For the cure of this affection lotions and all other kinds of dressings avail nothing; for at the bottom of the depression, hidden by overhanging folds of skin, there is a small fleshy polypus which has sprung from the scar of the fallen umbilical cord. By ligature of the growth the disease is at once cured.

